# Roles of H3K36-specific histone methyltransferases in transcription: antagonizing silencing and safeguarding transcription fidelity

**DOI:** 10.1007/s41048-018-0063-1

**Published:** 2018-08-29

**Authors:** Chang Huang, Bing Zhu

**Affiliations:** 1 National Laboratory of Biomacromolecules, CAS Center for Excellence in Biomacromolecules, Institute of Biophysics, Chinese Academy of Sciences, Beijing, 100101 China; 2 College of Life Sciences, University of Chinese Academy of Sciences, Beijing, 100049 China

**Keywords:** H3K36 methylation, Histone methyltransferase, SETD2, Ash1L, NSD

## Abstract

Histone H3K36 methylation is well-known for its role in active transcription. In *Saccharomyces cerevisiae*, H3K36 methylation is mediated solely by SET2 during transcription elongation. In metazoans, multiple H3K36-specific methyltransferases exist and contribute to distinct biochemical activities and subsequent functions. In this review, we focus on the H3K36-specific histone methyltransferases in metazoans, and discuss their enzymatic activity regulation and their roles in antagonizing Polycomb silencing and safeguarding transcription fidelity.

## Introduction

Chromatin features, including DNA modifications, histone modifications, histone variants, nucleosome occupation, and chromatin organization, regulate the regional accessibility of chromatin and thus modulate various chromatin-based biological processes, including replication, transcription, and repair. Histone acetylation and methylation at lysine residues are two of the most studied histone modifications, and they have interesting differences. Histone acetylation generally promotes active transcription by altering the positive charge at the lysine residues and the interactions between DNA and histone tails. Therefore, with few exceptions, the majority of histone acetyltransferases and deacetylases display broad substrate specificity and function for multiple lysine residues on various histones (Kouzarides 2007b). In contrast, histone lysine methylation does not change the charge at the histone tails and these methylated lysine moieties function by recruiting downstream reader proteins that are involved in gene activation or repression. The reader proteins generally display site-specificity due to the recognition of neighboring residues. This is probably the reason that histone methyltransferases and demethylases have co-evolved to have specific lysine site preferences (Kouzarides 2007a).

Histone H3K36 methylation is a hallmark of active transcription. Pioneer studies on budding yeast SET2, the first H3K36 methyltransferase (Strahl *et al*. 2002), have established a paradigm for the recruitment of SET2 and the function of H3K36 methylation: SET2 is recruited by Ser-2-phosphorylated Pol II during elongation to deposit H3K36me3, which functions as a docking site for the Rpd3S histone deacetylase complex to suppress cryptic transcription initiation (Venkatesh and Workman 2013). In metazoans, the characterization of multiple H3K36-specific methyltransferases has expanded the function of H3K36 methylation from transcription elongation to developmental gene regulation (Wagner and Carpenter 2012). In metazoans, SETD2 (also known as HYPB) is the sole enzyme responsible for H3K36me3 (Edmunds *et al*. 2008; Yuan *et al*. 2009); MES-4 (maternal-effect sterile 4) in *C. elegans* and *Drosophila* and its mammalian homologs, the NSD (nuclear receptor-binding SET domain) family proteins (including NSD1, NSD2, and NSD3), are the main contributors of global H3K36me2 (Bell *et al*. 2007; Bender *et al*. 2006; Kuo *et al*. 2011; Li *et al*. 2009b); Ash1 (absent, small and homeotic-1) in *Drosophila* and its mammalian homolog Ash1L (Ash1-like) can also produce H3K36me2, but they are limited more to the active Hox genes (An *et al*. 2011; Huang *et al*. 2017; Miyazaki *et al*. 2013; Schmahling *et al*. 2018; Tanaka *et al*. 2007; Yuan *et al*. 2011). MES-4 and Ash1 play critical roles in maintaining developmental gene expression, and the mutations and deregulation of human NSD family proteins and Ash1L are linked to various developmental diseases (Bennett *et al*. 2017; Rogawski *et al*. 2016; Wagner and Carpenter 2012). Moreover, the recent discoveries that “oncohistones” with H3K36M/I mutations can drive chondroblastoma tumorigenesis by inhibiting H3K36 methyltransferases and reprogramming the H3K36 methylation landscape further underscore the functional significance of H3K36 methylation (Fang *et al*. 2016; Lu *et al*. 2016). *In vitro*, H3K36 methylation directly inhibits PRC2, which catalyzes repressive H3K27 methylation (Schmitges *et al*. 2011; Yuan *et al*. 2011). *In vivo*, the genomic landscape of H3K36 and H3K27 methylation are anti-correlated (Gaydos *et al*. 2012; Lu *et al*. 2016; Papp and Muller 2006; Popovic *et al*. 2014; Yuan *et al*. 2011). These findings clearly underscore the role of H3K36 methylation in antagonizing Polycomb silencing. On the other hand, the distinct regulation of each H3K36-specific methyltransferase remains unclear. Here, we summarize the recent progress regarding the regulation of H3K36 methyltransferase activity and their roles in transcription. Notably, H3K36 methylation also participates in other aspects of chromatin events such as DNA repair and mRNA splicing, discussion of which is beyond the scope of this review, but has been reviewed by others (Fahey and Davis 2017; Li 2013; McDaniel and Strahl 2017; Wagner and Carpenter 2012).

## Auto-inhibition is a conserved regulatory mechanism of H3K36 methyltransferases

**Fig. 1:**
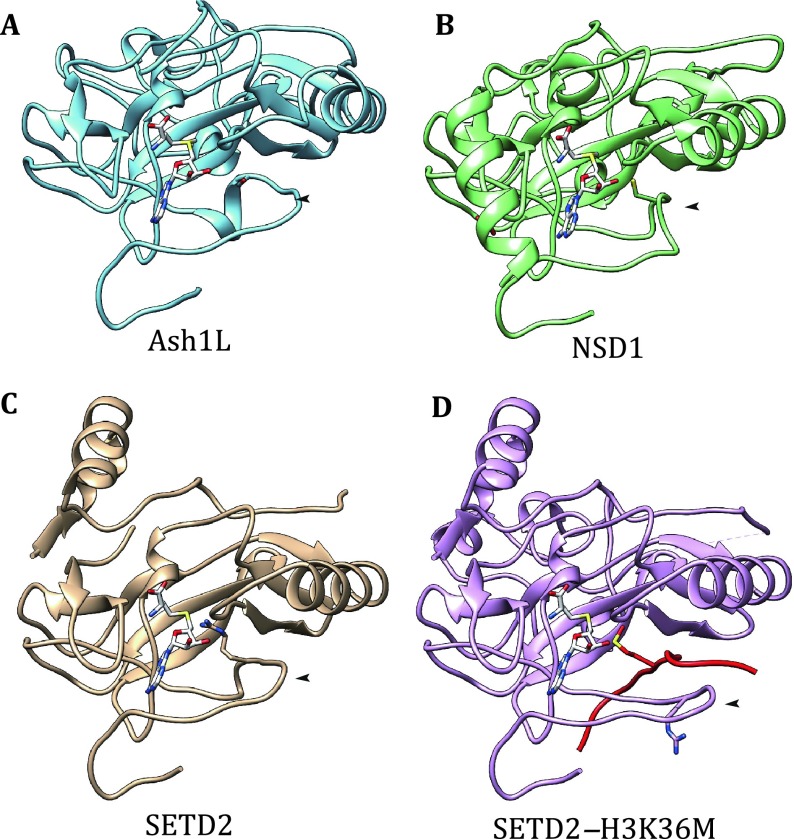
Auto-inhibitory loop is a shared feature of H3K36 methyltransferases that undergoes dynamic changes during catalysis. Structures of the catalytic domain of Ash1L (PDB code: 3OPE) (**A**), NSD1 (PDB code: 3OOI) (**B**), SETD2 (PDB code: 4H12) (**C**), and SETD2 bound with the H3K36 M peptide (PDB code: 5JJY) (**D**) are shown with arrowheads indicating the auto-inhibitory loop. Note that the side chain of S2259(Ash1L)/C211(NSD1)/R1670(SETD2) within the inhibitory loop occupies the positioning pocket of H3K36. In the SETD2–H3K36 M complex, R1670 flips out and allows the catalytic center to accommodate substrate binding

Studies reporting the characterization of the substrate specificity of H3K36-specific methyltransferases, especially dimethylases, displayed quite a number of disputes. After years of study, chromatin researchers have adopted the common belief that SETD2 is the sole enzyme responsible for H3K36me3, NSD family enzymes are the main contributors of H3K36me2, and Ash1/Ash1L is an enzyme governing H3K36me2 at specific regions (An *et al*. 2011; Dorighi and Tamkun 2013; Edmunds *et al*. 2008; Huang *et al*. 2017; Kuo *et al*. 2011; Li *et al*. 2009b; Miyazaki *et al*. 2013; Qiao *et al*. 2011; Streubel *et al*. 2018; Tanaka *et al*. 2007; Yuan *et al*. 2009, 2011). One likely explanation for the initial conflicting observations is that most H3K36-specific methyltransferases are nucleosome-specific enzymes; these methyltransferases are highly specific for H3K36 methylation at nucleosome substrates, but they display weak non-specific activities for non-nucleosomal histones (An *et al*. 2011; Byrd and Shearn 2003; Gregory *et al*. 2007; Li *et al*. 2009b; Tanaka *et al*. 2007; Yuan *et al*. 2009). The structural basis for the nucleosome-specific activities of H3K36-specific methyltransferases has not yet been resolved, and the exact molecular mechanisms of how nucleosomes confine the specificity and stimulate the catalytic activity of H3K36-specific methyltransferases remain unclear. Nevertheless, the structures of the catalytic domains of all three sub-types of H3K36 methyltransferases have been resolved, and interestingly, all of them share a conserved auto-inhibitory mechanism (Fig. 1) (An *et al*. 2011; Qiao *et al*. 2011; Zheng *et al*. 2012). A loop at the post-SET region occupies the binding channel for the histone H3 tail, thus blocking the access of lysine 36 to the catalytic center. Obviously, this loop must undergo a conformational change to remove this steric hindrance upon activation. Indeed, a half-opened conformation of the inhibitory loop was observed in SETD2, indicating the dynamic nature of this loop (Yang *et al*. 2016). Furthermore, when engaged with a K36M-mutated H3 peptide, which mimics the methylated product but cannot be released from the catalytic center, a fully opened state was observed (Fig. 1D) (Yang *et al*. 2016). In addition, biochemical studies suggest that nucleosomal DNA may act as an allosteric effector for NSD proteins (Li *et al*. 2009b), and computational docking and simulation suggest that the inhibitory loop of NSD1 may come into contact with the DNA and lead to lysine binding channel widening (Qiao *et al*. 2011). Therefore, it is reasonable to speculate that the engagement of nucleosome substrates with H3K36-specific methyltransferases may alter the auto-inhibitory loop to a conformation that favors H3K36 methylation catalysis.

Auto-inhibition is also an intrinsic characteristic of *E*(*z*), the catalytic subunit of the H3K27 methyltransferase PRC2. While *E*(*z*) alone is inactive (Antonysamy *et al*. 2013; Wu *et al*. 2013), engagement with two other core subunits of PRC2—EED and SUZ12—alters the configuration of its catalytic center and transforms it into an active conformation (Brooun *et al*. 2016; Jiao and Liu 2015; Justin *et al*. 2016). Therefore, another interesting speculation is that certain interaction partner(s) may induce a conformational change and activate H3K36-specific methyltransferases. SETD2 and Ash1/Ash1L have stable interaction partners (Huang *et al*. 2017; Schmahling *et al*. 2018; Yuan *et al*. 2009). In human cells, SETD2 interacts stably with HnRNP-L, which facilitates H3K36me3 deposition *in vivo*. However, HnRNP-L does not stimulate the enzymatic activity of SETD2 *in vitro*, which suggests that HnRNP-L does not function as a catalytic activator of SETD2 (Yuan *et al*. 2009). Recently, we and others demonstrated that *Drosophila* Ash1 and human Ash1L form stable complexes with Mrg15 (or human Morf4L1/2) and Nurf55 (or human RbAp46/48) (Huang *et al*. 2017; Schmahling *et al*. 2018). Interestingly, MRG domain-containing proteins, including Mrg15 and human Morf4L1/2, stimulate the catalytic activity of Ash1/Ash1L significantly. It will be interesting to determine whether Mrg15 activates Ash1 by inducing a conformational change that eliminates the blockage of the catalytic center by the auto-inhibitory loop of Ash1. Although the exact mechanism of induction remains to be determined, this is the first case of allosteric activation directed by an interaction partner among H3K36-specific methyltransferases.

In addition to the positive regulation of catalytic activities, all three sub-types of mammalian H3K36-specific methyltransferases are inhibited directly by H2A ubiquitination (Yuan *et al*. 2013). Whether such negative regulation involves the stabilization of the auto-inhibitory loop is an interesting question for future exploration.

The physiological roles of this auto-inhibition remain unclear, but this process has been proposed to protect enzymes from hyperactivation (Wang *et al*. 2015). An intriguing hypothesis is that auto-inhibition and its bypass are ideal interfaces for additional regulation, such as signaling events. Thus, the catalytic process of H3K36-specific methyltransferases is subject to intricate regulations directed by both intrinsic and external mechanisms.

## Diversified chromatin recruitment of H3K36-specific methyltransferases assigns distinct physiological function to H3K36 methylation

In *Saccharomyces cerevisiae*, SET2 generates all forms of H3K36 methylation, with trimethylation being the primary effective mark; however, H3K36me2 can work as efficiently as H3K36me3 in recruiting Rpd3S and suppressing cryptic initiation (Li *et al*. 2009a), suggesting indiscriminate roles of di- and trimethylation under this context. In metazoans, ChIP-sequencing showed that H3K36me2 demarcates chromatin differently from H3K36me3: within genic regions, H3K36me2 is preferentially enriched proximal to the transcription start sites and gradually decays downstream into the H3K36me3-enriched 3′ region; in addition, the large amount of H3K36me2 spread across intergenic regions implies a role at distal regulatory elements (Kuo *et al*. 2011). Although SETD2 preserves the capacity to generate all three states of methylation, SETD2 depletion affects only H3K36me3, not H3K36me1/2, at the bulk level (Edmunds *et al*. 2008; Yuan *et al*. 2009), indicating the specific assignment of trimethylation for transcription elongation. Moreover, the evolvement of several methyltransferases specific for H3K36me2 and the fact that mutations and deregulation of these enzymes cause varied developmental diseases further suggest that H3K36me2 may have a function distinct from that of H3K36me3. In convergence, these di-methyltransferases are recruited to chromatin differently from SETD2 (see discussion below), which expands the H3K36 methylation territory and diversifies the function of H3K36 methylation. H3K36 also exists in mono-methylated state. However, as an intermediate product of methylation reactions catalyzed by the aforesaid di- and tri-methyltransferases, the chromatin distribution and functional role of H3K36me1 remain poorly characterized.

### SET2/SETD2 and H3K36me3: recruited by elongating Pol II to safeguard transcription fidelity

As mentioned, in *Saccharomyces cerevisiae*, SET2 is recruited by the Ser-2-phosphorylated C-terminal domain (CTD) of elongating RNA polymerase II, and it deposits H3K36 methylation at the gene bodies of active genes (Krogan *et al*. 2003; Li *et al*. 2002, 2003; Xiao *et al*. 2003). Successive transcription may cause histone hyperacetylation in gene bodies, which allow cryptic transcription initiation. To prevent such deleterious events, transcription elongation-coupled H3K36 methylation serves as a docking site for the histone deacetylase complex Rpd3S, which restores the repressive chromatin environment following Pol II passage to prevent cryptic transcription initiation (Carrozza *et al*. 2005; Joshi and Struhl 2005; Keogh *et al*. 2005; Li *et al*. 2007).

Transcription elongation-coupled SETD2 recruitment and H3K36me3 deposition are conserved in mammals, as well as the role of H3K36me3 to prevent aberrant transcription initiation; however, whether the repressive environment promoted by H3K36me3 depends on histone deacetylases remains unexplored. Intriguingly, in mammals, the PWWP domain containing *de novo* DNA methyltransferases DNMT3A/B is recruited by H3K36me3 to methylate intragenic DNA, which may, in turn, recruit methylated DNA-binding proteins and histone deacetylases (Jones 2012; Neri *et al*. 2017), to safeguard transcription initiation. Indeed, mouse ES cells lacking DNA methylation exhibited intragenic transcription initiation (Neri *et al*. 2017).

Overall, SETD2 deposits H3K36me3 in gene bodies to recruit downstream machineries to restore the non-permissive chromatin state following Pol II passage and to maintain transcription fidelity at the genome level.

### H3K36me2 methyltransferases and H3K36me2: demarcating active chromatin by antagonizing silencing

Loss-of-function studies in multiple species, including *C. elegans*, *Drosophila*, and mammalian cells, indicate that NSD family proteins are responsible for bulk chromatin H3K36me2 levels (Bell *et al*. 2007; Bender *et al*. 2006; Kuo *et al*. 2011), implying the widespread distribution of these enzymes.

In *C. elegans*, MES-4, a homolog of mammalian NSD proteins, is vital for germ cell viability (Bender *et al*. 2006) and is highly abundant in H3K36me2-enriched autosomes, but not in the H3K27me3-enriched X chromosome in germline cells (Bender *et al*. 2006; Fong *et al*. 2002). Moreover, an MES-4 ChIP-chip analysis of early embryos revealed that MES-4 is distributed around the gene bodies of approximately 20% of genes, among which germline-specific genes are highly enriched (Rechtsteiner *et al*. 2010). MES-4 signals arise near TSS regions, peak proximally, and gradually decrease towards the 3′ end of gene bodies, correlating well with the pattern of H3K36me2 at genic regions (Rechtsteiner *et al*. 2010).

In *Drosophila*, MES-4 is also enriched at the 5′ end of target genes (Bell *et al*. 2007). Importantly, dMES-4 is recruited by an insulator-binding protein to promote the transcription of flanking genes by antagonizing the spread of H3K27 methylation from nearby regions (Lhoumaud *et al*. 2014); this finding revealed the functional role of the MES-4/NSD family of enzymes at the intergenic cis-regulatory regions.

In mammals, there are three NSD family proteins, and their chromatin localization has not been thoroughly analyzed. Knocking down NSD1 in ESC cells reduces H3K36me2 levels throughout the genome—at gene promoters, gene bodies, and intergenic regions (Streubel *et al*. 2018). NSD2 localizes to active transcripts, with a greater preference for elongating regions and distal regulatory regions (Ram *et al*. 2011). Consistently, in multiple myeloma, t(4;14) chromosomal translocation resulting in NSD2 overexpression led to the aberrant accumulation of H3K36me2 at both the intragenic and intergenic regions, supporting the widespread targeting of NSD2 (Kuo *et al*. 2011; Popovic *et al*. 2014). The distribution of full-length NSD3 has not been reported, but a short isoform of NSD3 possessing the PWWP domain localizes preferentially to enhancers and promoters (Shen *et al*. 2015). Importantly, in chondroblastomas, recurrent H3K36M mutations reprogrammed the transcriptome through inhibiting and sequestering H3K36 methyltransferases, resulting in a global reduction in H3K36 methylation; in this process, intragenic and intergenic H3K36me2 were mediated largely by NSD proteins (Fang *et al*. 2016; Lu *et al*. 2016). These findings further underscore the physiological significance of the broad targeting of NSD proteins.

NSD proteins can interact with nuclear receptors, suggesting their recruitment by transcription factors (Huang *et al*. 1998); however, the general targeting mechanism remains unknown, especially for the intergenic regions. Notably, NSD proteins contain multiple chromatin reader modules, including the PWWP and PHD domains, which may contribute to the spread of NSD proteins (He *et al*. 2013; Sankaran *et al*. 2016). Overall, the NSD family of proteins targets numerous genes involved in many development pathways and extensive intergenic regions.

Different from the MES-4/NSD family of enzymes, the other H3K36me2-specific methyltransferase Ash1 functions as a trithorax protein in *Drosophila* to maintain the expression of a small collection of developmental genes, the HOX genes. ChIP analysis showed that Ash1 is distributed throughout its target genes (Huang *et al*. 2017; Schwartz *et al*. 2010). A subset of cis-regulatory elements in the *Drosophila* genome that can recruit Trithorax/Polycomb group proteins was identified and defined as Trithorax/Polycomb response elements (TRE/PRE) (Ringrose and Paro 2007). Transgenic TRE/PREs can recruit Trithorax/Polycomb proteins ectopically to maintain the active/repressive states of reporter genes, underscoring their significance in Trithorax/Polycomb recruitment. Importantly, the sequences of TRE/PREs are the same in different cell types, but Trithorax and Polycomb proteins, including Ash1, have different locations in each cell type, suggesting additional regulators beyond DNA sequences. Moreover, Ash1 cooperates with other Trithorax group members to maintain Hox gene expression, among which Trithorax and Kismet may directly promote the chromatin recruitment of Ash1: Ash1 and the N-terminus of Trithorax display an interdependency on chromatin localization (Schwartz *et al*. 2010); knocking down kismet, a CHD family chromatin remodeler, greatly reduces the chromatin retention of Ash1 (Srinivasan *et al*. 2008), suggesting that chromatin accessibility directed by a chromatin remodeler also affects Ash1 recruitment. Given that the Ash1 protein also contains multiple chromatin binding domains, including Bromo, BAH, and PHD, and that its partner protein Mrg15 also contains a Chromo domain that recognizes H3K36 methylation, it is natural to expect that the recognition of a combination of histone modifications will contribute another regulatory layer to Ash1 recruitment. Taken together, DNA elements, transcription factors, and the chromatin environment may function coordinately to shape the binding profile of Ash1. The recruitment of Ash1L in mammalian systems is not well studied, and PREs/TREs are not defined in mammals. Despite this, Ash1L also regulates the HOX genes in mammals, indicating the conservation of the recruitment and function of Ash1L (Miyazaki *et al*. 2013).

Overall, NSD proteins and Ash1L are associated with active transcription, the malfunction of which leads to gene inactivation. Mechanistically, H3K36me2/3 inhibits the catalytic activity of PRC2 (Schmitges *et al*. 2011; Yuan *et al*. 2011), and the mutually exclusive distribution of H3K36 methylation and H3K27 methylation along chromatin has been observed in many biological systems (Gaydos *et al*. 2012; Lu *et al*. 2016; Papp and Muller 2006; Popovic *et al*. 2014; Yuan *et al*. 2011). While the anti-silencing mechanisms of NSD proteins and Ash1L are similar, it would be of great interest to uncover their distinct recruitment mechanisms, which will help in the understanding of the biological impact of these distinct enzymes.

## Summary and perspectives

**Fig. 2:**
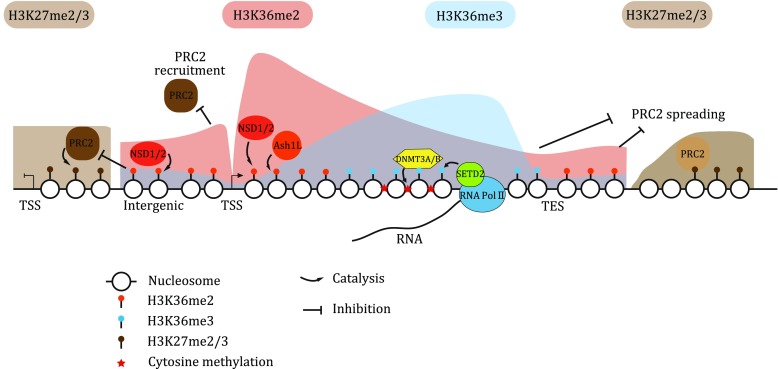
Working model of H3K36-specific methyltransferases and H3K36 methylation in mammalian gene transcription regulation

In higher eukaryotes, in addition to its conservative role in transcription elongation, H3K36 methylation has an additional function: anti-silencing. In addition to targeting certain developmental genes, PRC2-mediated transcription silencing through H3K27 methylation seems to establish and maintain a default repressive state for most of the inert genome. PRC2 may be recruited initially by cis-elements and then propagate along chromatin through a reinforced spreading mechanism (Wang *et al*. 2018). On the other hand, active genes and regulatory regions have adapted multiple mechanisms, including installation of H3K4 and H3K36 methylation, as well as an open chromatin status, which are all repulsive substrates for PRC2 catalysis, to overcome the silencing effect (Schmitges *et al*. 2011; Yuan *et al*. 2011, 2012). Both H3K36me2 and H3K36me3 can inhibit PRC2 activity efficiently *in vitro* (Schmitges *et al.* 2011; Yuan *et al.* 2011). However, they may antagonize PRC2 in different ways *in vivo*: PRC2 generally targets promoters and enhancers, but not gene bodies, for the initiation of silencing, so protecting these cis-elements with H3K36me2 via NSD/Ash1L may abolish the initial recruitment of PRC2; in addition, the general H3K36me2 enrichment and the gene body-enriched H3K36me3 at active genes may inhibit the spread of H3K27 methylation from adjacent regions (Fig. 2). Taken together, we propose that NSD/Ash1L-mediated H3K36me2 may act as the primary effector to actively repel PRC2 silencing at developmental genes, thus maintaining their expression.

Although we attempt to dissect the specific roles of each H3K36 methyltransferase and the different forms of H3K36 methylation to clarify their intrinsic biological functions, they often work in concert and affect each other in many cases. For instance, the transcription of genes maintained by the NSD family proteins/Ash1L will certainly upregulate SETD2 and H3K36me3, and the loss of the NSD family proteins/Ash1L, resulting in transcription inactivation, will surely cause the loss of SETD2 and H3K36me3.

Now two decades old, our knowledge of H3K36-specific methyltransferases and H3K36 methylation is still expanding. The links between the deregulation of H3K36-specific methyltransferases and various biological outcomes in diseases have not been fully established. Hopefully, a thorough understanding of the mechanism and function of H3K36-specific methyltransferases will help to pave the way for designing specific and rational targeting strategies for these diseases in the future.
